# A Potential Nanofiber Membrane Device for Filling Surgical Residual Cavity to Prevent Glioma Recurrence and Improve Local Neural Tissue Reconstruction

**DOI:** 10.1371/journal.pone.0161435

**Published:** 2016-08-22

**Authors:** Daoxiang Huang, Chao Lin, Xuejun Wen, Shuying Gu, Peng Zhao

**Affiliations:** 1 The Institute for Translational Nanomedicine, Shanghai East Hospital, Institute of Biomedical Engineering and Nanoscience, Tongji University School of Medicine, Shanghai, People's Republic of China; 2 Key Laboratory of Advanced Civil Engineering Materials Ministry of Education, School of Material Science and Engineering, Tongji University, Shanghai, People's Republic of China; 3 Institute of Nano and Bio-Polymeric Materials, School of Material Science and Engineering, Tongji University, Shanghai, People's Republic of China; Universita del Salento, ITALY

## Abstract

This study aims to develop a novel device with nanofiber membrane capable of sustained release of temozolomide (TMZ) and neuron growth factor (NGF). An improved bio-availability of TMZ and NGF in surroundings proximal to the device was expected to be attained for a prolonged period of time. The device was developed by integrating TMZ-doped polycaprolactone (PCL) nanofiber (TP) membrane and NGF-coated PCL (NGFP) membrane using sodium alginate hydrogel. TP was prepared by direct electrospinning of TMZ/PCL. NGFP membrane was developed by layer-by-layer assembling technology. The incorporation of TMZ-doped nanofiber and NGFP nanofiber in the device was confirmed by scanning electron microscopy. The number of NGF layer in NGF-coated PCL membrane could be readily measured with energy spectrum analysis. The *in vitro* release study showed that TP-NGFP-TP membrane could efficiently liberate TMZ to inhibit the growth of C6 glioma cells, and sufficient NGF to induce the differentiation of PC12 neuron cells over four weeks. Such TP-NGFP-TP membrane device can be employed as a tampon to fill up surgical residual cavity and afford residual glioma removal, structural support, hemostasis, and local neural tissue reconstruction in the surgical treatment of glioma. The study opens a horizon to develop multifunctional biomaterial device for maximized glioma treatment efficacy.

## Introduction

Gliomas have been the most severe brain tumors [1]. The incidence of gliomas is relative to 80% of malignant brain tumors [2]. Surgical resection is the key procedure of glioma treatment. Surgical residual cavity and its proximal surroundings are usually the breeding sites of recurrence or metastasis arisen from residual tumor cells due to robust invasiveness property of glioma [3]. Inhibition of residual glioma cells after surgery is highly relied on adjuvant radiotherapy or chemotherapy [4]. Temozolomide (TMZ) is an oral chemotherapeutic medicine for glioma [5], resulting in glioma cell apoptotic death and local neural tissue destruction. However, the efficacy of TMZ is far less satisfactory, being attributable to its low local bio-availability resulted from the blood brain barrier [6] and drug resistance associated to prolonged treatment for its low bio-availability [7, 8].

Undoubtedly, drug carriers could improve the utilization level of chemotherapeutics. Particularly, at the surgical site for a sufficient period of time, they would enhance the removal of residual glioma and prevent its recurrence and metastasis. Some articles have focused on the construction of drug delivery systems against glioma. Feng *et al*. developed PEG-PLA based nanoparticles loaded with paclitaxel. The nanoparticles could be successfully concentrated in the glioma area through the modification of a peptide on the surface of the nanoparticles [9]. Ju *et al*. prepared targeting liposomes incorporated with epirubicin and celecoxib for the treatment of glioma and destruction of vasculogenic mimicry channels to prevent glioma recurrence [10]. These studies indicated that nanoparticles could target markers and transporters on the surface of glioma cells, which are helpful to enhance utilization level of chemotherapeutics and reduce side effects. However, nanoparticle-based drug delivery systems indeed suffer from off-target effect. Furthermore, the burden on the patients with glioma is not significantly improved, because a long-term medication is necessary.

Nanofiber technology has recently shown compelling advancement in drug loading and release, offering an opportunity to develop innovative formula to improve local TMZ delivery in glioma treatment. Nanofiber is a kind of nanoscale fiber produced by electrospinning. Over the past two decades, a lot of nanofibers made of collagen, polycaprolactone (PCL) and polylactide have been used as implant devices for local drug delivery in postsurgical treatment [11]. Electrospinning nanofibers as promising drug delivery carriers were explored for their improved capacity on drug loading and release [12]. Kaplan *et al*. reported on cisplatin-loaded nanofiber meshes which can enhance median recurrence-free survival in the murine model [13]. Liu *et al*. prepared multilayer polylactide nanofiber mats doped with oxaliplatin and the recurrence was significantly retarded [14]. Recently, Ni *et al*. prepared polypropylene carbonate nanofibers loaded with paclitaxel and TMZ [15]. The authors showed that the co-delivery of paclitaxel and TMZ on site was highly efficient to inhibit *in vitro* growth of glioma cells, indicating great possibility of the nanofibers as drug delivery devices for glioma therapy.

**Fig 1:**
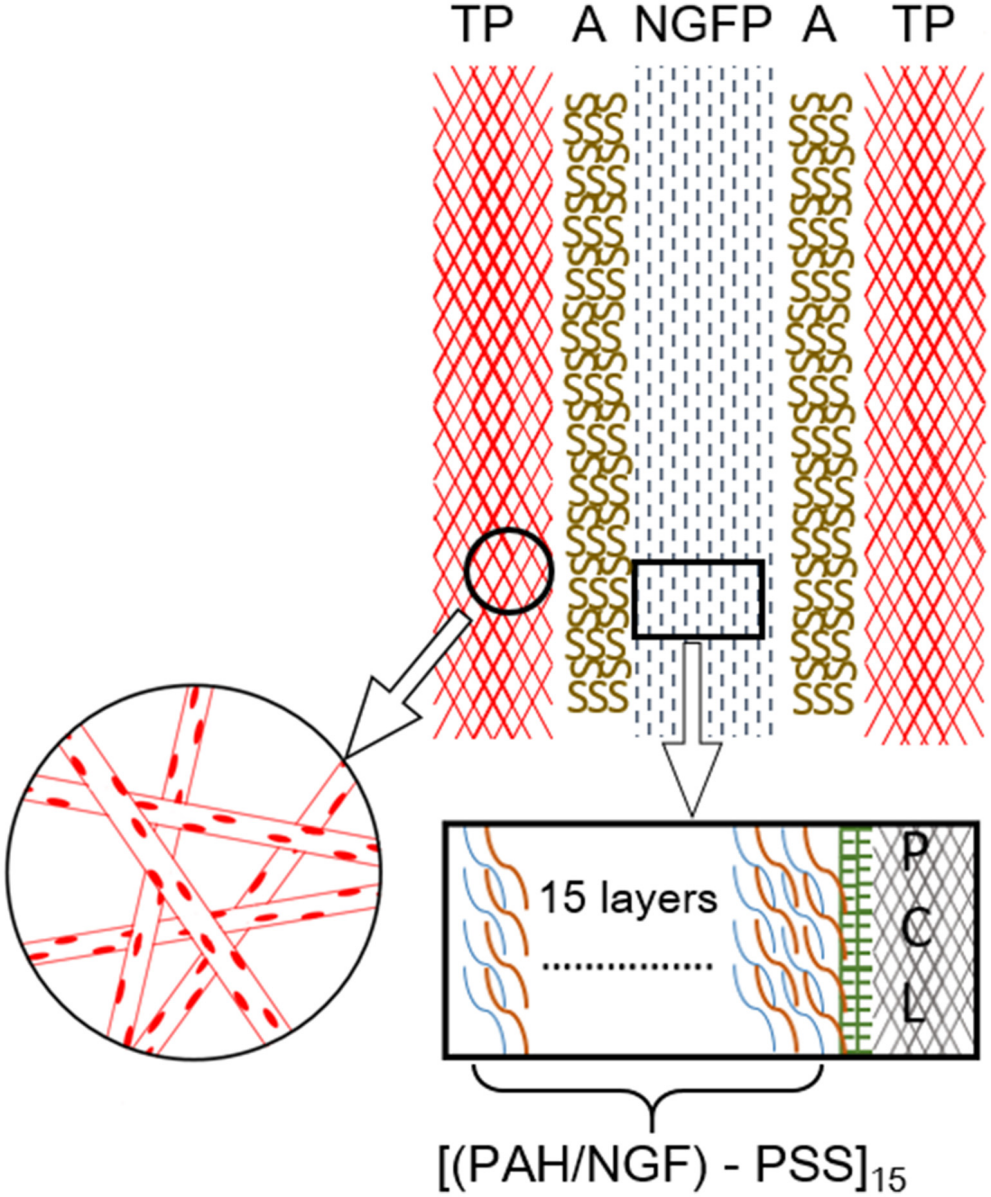
An illustration of dual-function device designed for dual-stage release of TMZ and NGF. Notes: TP means TMZ-doped PCL membrane; A means sodium alginate hydrogel; NGFP means NGF-coated PCL membrane.

TMZ has noticeable side effect on the local neural tissue destruction [16], causing the delay in recuperating from surgical treatment of glioma. From clinical point of view, it is preferable to develop a surgical residual cavity tampon capable of improving residual tumor removal and meanwhile reducing side effects on normal neural tissue and facilitating nerve regeneration. To our best knowledge, such a multi-functional technology has not yet been reported. Those TMZ-loaded nanofibers reported for glioma chemotherapy are unable to improve neuron cell growth. In view of this regarding, a dual-function device, integrating the removal of residual glioma cells and the reconstruction of local neural tissue, was designed in this study. As shown in Fig 1, the device comprises an outer layer of TMZ-loaded PCL-based nanofiber (denoted as TP) membrane and an inner layer of neuron growth factor (NGF)-coated PCL (denoted as NGFP) membrane obtained by layer-by-layer assembly of PSS and PAH/NGF on the surface of PCL membrane. We hypothesized that the TP membrane could serve for sustained TMZ release against glioma and the NGFP membrane could stabilize and deliver NGF for neuron cell proliferation and differentiation. The present study delineates the fabrication and characterization of such TP-NGFP-TP membrane device in terms of its morphology and drug release profile. The *in vitro* effects of this device on glioma cell growth and neuron cell differentiation were also evaluated.

## Materials and Methods

### 2.1 Materials

Poly(ε-caprolactone) (PCL, M_w_ = 80kDa), polyethlenimine (PEI, 50 wt% in water solution, M_w_ = 50kDa), poly(allylamine hydrochloride) (PAH, M_w_ = 15kDa), poly (sodium 4-styrenesulfonate) (PSS, M_w_ = 70kDa) and sodium alginate were purchased from Sigma-Aldrich (USA). NaCl, CaCl_2_, dichloromethane (DCM) and dimethyl sulfoxide (DMSO) were ordered from Sinopharm Chemical Reagent Co. (PR China). Temozolomide (TMZ) and nerve growth factor (NGF) were kindly gifted by Changhai Hospital of Shanghai. FITC-labeled rabbit anti-human IgG (H+L)(0.75 mg/ml, 50 wt% glycerin + 50 wt% ultrapure water) and 4% paraformaldehyde were purchased from DingGuo ChangSheng Biotechnology Co. (Beijing, PR China). Pancreatin, Dulbecco’s modified Eagle’s medium (DMEM), RPMI-1640 medium, and penicillin/streptomycin were purchased from GIBCO (USA). Anti-β III tubulin antibody [2G10] (ab78078) and secondary antibody goat anti-mouse IgG H&L (Alexa Fluor 488^®^, ab150113) were purchased from Abcam^®^ (UK). DAPI working solution was purchased from Beyotime Institute of Biotechnology (Jiangsu, PR China). Triton X-100 was purchased from Aladdin (Shanghai, PR China).

### 2.2 Fabrication of TMZ-loaded PCL nanofiber membrane (TP membrane)

A series of spinning solutions were prepared by mixing 14 wt% of PCL solution in DCM and 2 wt% of TMZ solution in DMSO at varied weight ratio 70/30, 60/40 and 50/50, respectively. The solutions were homogenized in an ultrasonic cleaner and then placed in a glass syringe (15 mL) equipped with a 7-gauge metal needle (0.4 mm of internal diameter). Nanofiber membrane was prepared using an electrospinning device on an aluminum plate as a collector under the voltage of 15kV supplied by a high voltage research device (ZS-60kV/2mA, Rixing Electric Inc. Shanghai, China). The distance between the needle tip and the plate was set at 20 cm and the solution feeding rate was set at 0.7 mL/h driven by a syringe pump (LSP01-1A, LongerPump Inc., Baoding, China). The electrospinning device was kept at 40°C and 30% humidity. The nanofiber membrane was pulled off from the plate after solvent evaporation and stored at room temperature before use.

### 2.3 Fabrication of NGF-coated PCL nanofiber membrane (NGFP membrane)

NGFP membrane was obtained by LBL technology on PCL membrane. All the polyelectrolytes were dissolved in ultrapure water. In brief, PCL membrane was immersed in PEI solution (5 mg/mL) for 30 min. The membrane was gently flushed with ultrapure water to remove non-absorbed PEI, then, immersed in PSS solution (1 mg/mL) for 10 min and flushed with ultrapure water. The membrane was finally immersed in PAH solution (1 mg/ml) containing NGF (1 μg/mL) for 10 min and flushed ultrapure water. The immersing in PSS and PAH solution was repeatedly performed for 15 times. The resulting NGFP membrane was cut into 2×2 cm^2^ and stored at 4°C before use.

NGF release behavior of NGFP membrane was indirectly determined by observation of IgG release behavior of PCL (IgGP) nanofiber membrane coated with FITC labeled IgG for its availability, the technical readiness to measure the fluoresce labeled IgG and reliable correlation among proteins in release studies. In parallel to developing NGFP membrane, IgG PCL (IgGP) nanofiber membrane coated with FITC labeled IgG was prepared. Briefly, PCL membrane was coated by immersing in positively-charged IgG/PSS solution (1 mg/mL) and negatively-charged PAH solution (1 mg/mL) for 15 times. IgG concentration in IgG/PSS solution was 1 μg/ml. IgGP membrane was cut into 2×2 cm^2^ and stored at 4°C before use.

### 2.4 Construction of a multi-layer sandwich device integrated with TP and NGFP membranes

A multi-layer membrane device was constructed by overlaying TP/NGFP/TP membrane sandwiches glued with sodium alginate hydrogel. In brief, TP membrane with solvent in a ratio of 70/30 was firstly prepared according to the aforementioned protocol. Then, NGF membrane was coated with sodium alginate solution (75 mg/ml) and three-layer membranes were overlaid in the sequence of TP/NGF/TP. Finally, the sandwich device was immersed in CaCl_2_ solution (0.2 g/mL) over 10 min to form alginate hydrogel. After gently flushing twice with water, the sandwich membrane device was stored at 4°C for further use.

### 2.5 Characterization of nanofiber membranes

#### 2.5.1 Morphology and energy analysis

Morphologies of PCL, TP and NGFP nanofiber membranes were observed by scanning electron microscopy (SEM) (Vega3, Tescan Co. Ltd., Brno, Czech Republic and S-4800, hitachi, Japan) at a voltage of 5kV. Energy analysis of NGFP membrane was performed by SEM supplied with energy disperse plug-in (Esprit 1.8, Bruker AXS Microanalysis GmbH Berlin, Germany) at a voltage of 20kV. Average diameter of nanofiber and its distribution were estimated by Image J software.

#### 2.5.2 Membrane mechanical property

Mechanical strengths of PCL and TP nanofiber membranes were measured by stretching the samples in opposite directions by computer controlled single axial tensile machine (XJ810-50N, Xiangjietest, Shanghai, P.R.China). The samples for mechanical tests were pre-treated by cutting into 40 mm×20 mm (H×W) and measured at a constant speed (1 mm/min) to simulate static stretching.

#### 2.5.3 Nanofiber membrane surface property

Surface property of PCL and NGFP nanofiber membranes was investigated by Sessile drop method using a contact angle apparatus (JC2000C1, Powereach, Shanghai, P. R. China). Briefly, the membranes were cut into 1×1 cm^2^. A micro-syringe was used to give one drop of water (25 μL) onto the surface of the membranes in testing. Contact angle was then measured.

#### 2.5.4 Drug content of TP membrane

TMZ content of TP membrane was determined by measuring TMZ extracted in a solvent with ultraviolet spectrum analyzer (Cary 50, Varian medical systems Co. Ltd., USA). In brief, 5 cm^2^ of TP membrane was dissolved into DCM (1 mL), then diluted with phosphate buffer (pH7.4, 5 mL). The buffered solution was analyzed at the wavelength of 329 nm. TMZ content was calculated based on the standard curve developed from a group of TMZ standard solutions and expressed with the unit of mg/cm^2^.

#### 2.5.5 Membrane release property

TMZ release behavior from TP membrane was investigated using ultraviolet spectrum analyzer. Briefly, 5 cm^2^ of TP membrane was incubated in a tube containing phosphate buffer saline (PBS, pH7.4, 10 mL). The tube was kept in an incubator at 37°C. At various time intervals ranging from 1 to 30 days, TMZ content in the buffer was determined by UV analysis. The membrane was moved into fresh PBS (10 mL) for subsequent tests. Each group have 5 parallels to avoid individual difference.

The release behavior of IgGP membrane was investigated by determining the content of FITC-labeled IgG with a fluorescence meter at excitation wavelength of 485 nm and emission wavelength of 538 nm, respectively. Briefly, 3 cm^2^ of IgGP membrane was incubated in a tubes containing PBS (3 mL). The tube was kept in an incubator at 37°C. At different time intervals from 2 to 30 days, the PBS solution in the tube was used for determining IgG content based on a standard curve developed from a series of standard FITC-IgG solutions. Each group has 5 parallels.

### 2.6 The *in vitro* effects of TP-NGFP-TP device on glioma cell proliferation and neuron differentiation

C6 glioma cells or PC12 neuron cells were separately cultured in DMEM or RPMI-1640 medium supplied with 10% FBS and 1% Penicillin-streptomycin, and incubated at 37°C in air containing 5% CO2. The *in vitro* effect of TP-NGFP-TP device on proliferation of C6 cells was investigated. In brief, C6 cells (5×10^3^ cells/well) were cultured in a 96 well plate for at least 24 h. TP-NGFP-TP device was cut into 5 mm×5 mm and incubated in PBS (37°C, pH = 7.4) for 0, 7 and 14 days. After pretreatment, the membranes device was co-incubated with the cells for 1, 2 and 3 days. Cell number was determined with a Cell Counting Kit-8 (CCK-8, Dojindo Molecular Technologies, Inc., Japan), according to the manufacturer’s protocol. Optical density (O.D.) at 450 nm was measured by microplate reader (multiskan MK3, Thermo Fisher Scientific, USA). The data were shown as average±standard deviation (n = 5). Each group has 5 parallels to avoid individual difference.

The *in vitro* effect of TP-NGFP-TP device on differentiation of PC12 cells was investigated. In brief, PC12 cells (2×10^4^ cells/well) were cultured in a 24 well plate for 24 h. TP-NGFP-TP device was pre-incubated in PBS (37°C, pH = 7.4) for 2 weeks. NGFP membrane or treated TP-NGFP-TP device at the size of 1 cm×1 cm was co-incubated with the cells for 1, 3 and 5 days. The effect on PC12 cell growth was evaluated by observing promotion of PC12 neuron cells differentiation under optical microscope after 24 h culture.

### 2.7 Immunofluorescence staining of β-tubulin III

PC12 cells were fixed with 100% methanol for 5 min then rinsed with PBS (pH = 7.4). Cells were treated with 4% paraformaldehyde for 20 min then rinsed with PBS. After that, Cells were immersed in 0.1% Triton X-100 for 30 min then rinsed with PBS. After incubated in 1% BSA for 30 min, cells were incubated with anti-β tubulin III antibody dilution (1 μg/ml) overnight at 4°C. Then, cells were rinsed with PBS and incubated with secondary antibody goat anti-mouse IgG H&L dilution (1:500) for 1h at room temperature. Finally, cells were rinsed with PBS and stained with DAPI working solution at room temperature for 5 min. Result were observed by Cytation 3 cell imaging multi-mode reader.

## Results and Discussion

### 3.1 Preparation and Characterization of NGFP membranes

**Fig 2:**
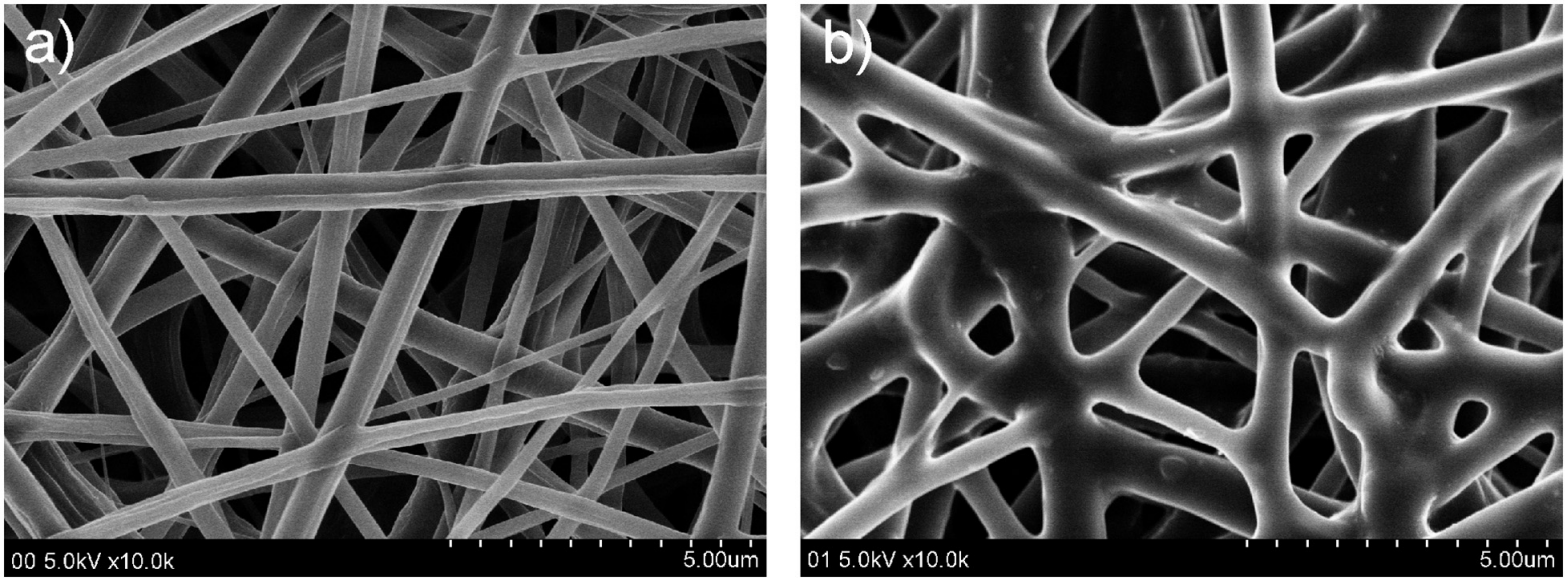
SEM images of NGF membrane. **PCL membrane is used as a control**. a) PCL membrane; b) NGFP membrane.

**Fig 3:**
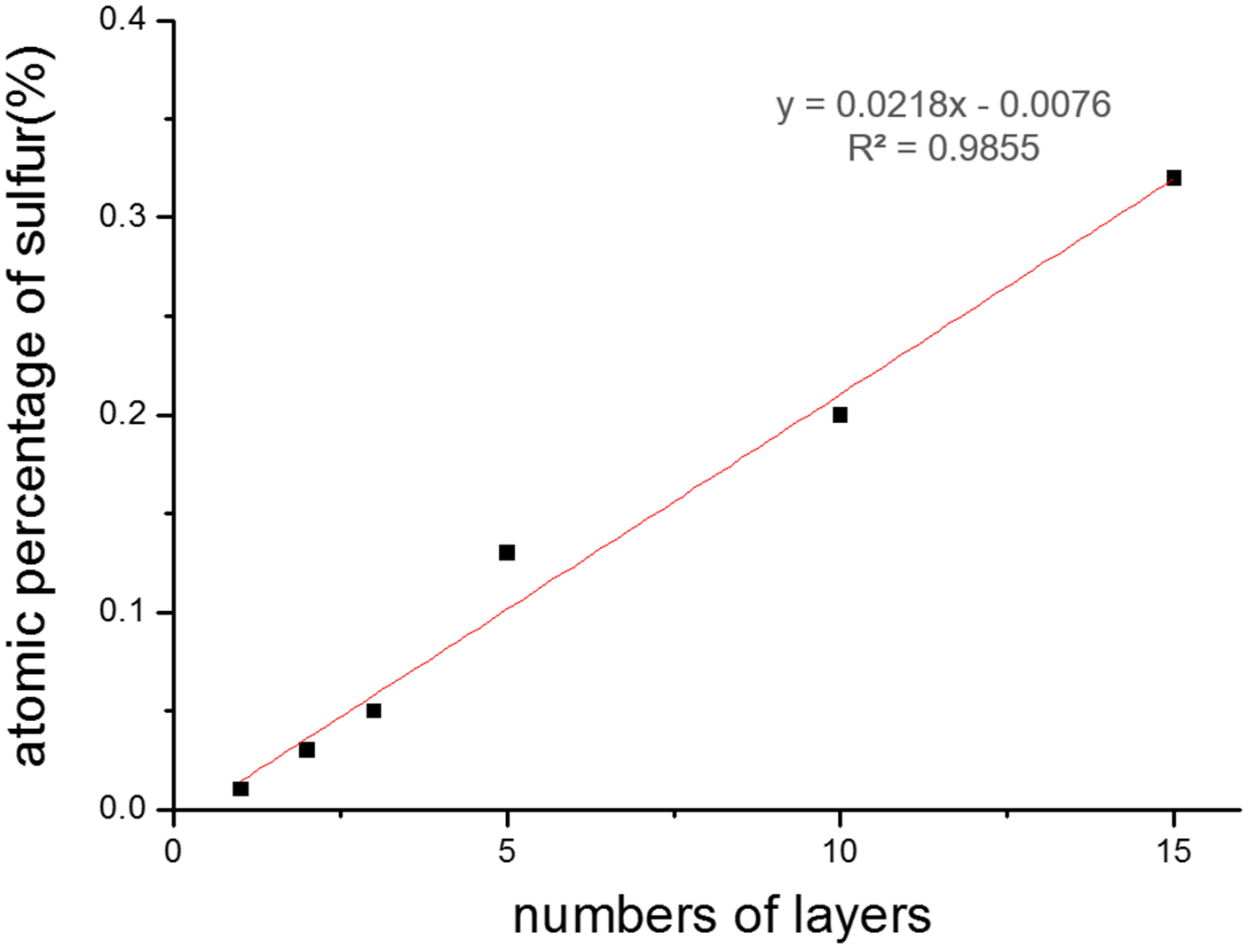
Surface sulfur content of NGFP nanofiber membrane by energy disperse.

In order to incorporate NGF into PCL nanofiber membrane, we used layer-by-layer (LBL) method to prepare multiple-layer NGF-coated PCL (NGFP) membrane through electrostatic interaction between negatively-charged PSS and positively-charged PAH/NGF mix. Fig 2 indicates typical SEM images of NGFP membrane having 15-layer NGF, where the PCL nanofibers showed a smooth surface. It should be noted that the LBL-treated PCL membranes remained nanofiber’s original morphology and were favorable for cell proliferation. The NGF treated nanofibers were bigger in diameter than native PCL (680.0±240.0 *vs*. 396.0±141.3). Energy disperse analysis of NGFP membranes with different number of layers showed that sulphur content was increased over 3 to 20 times (Fig 3 and Fig A in S1 File). The increment tendency was approximately linear, indicating that LBL assembling process was successful. The amount of NGF to assemble can be regulated by alteration of accumulated layers.

**Fig 4:**
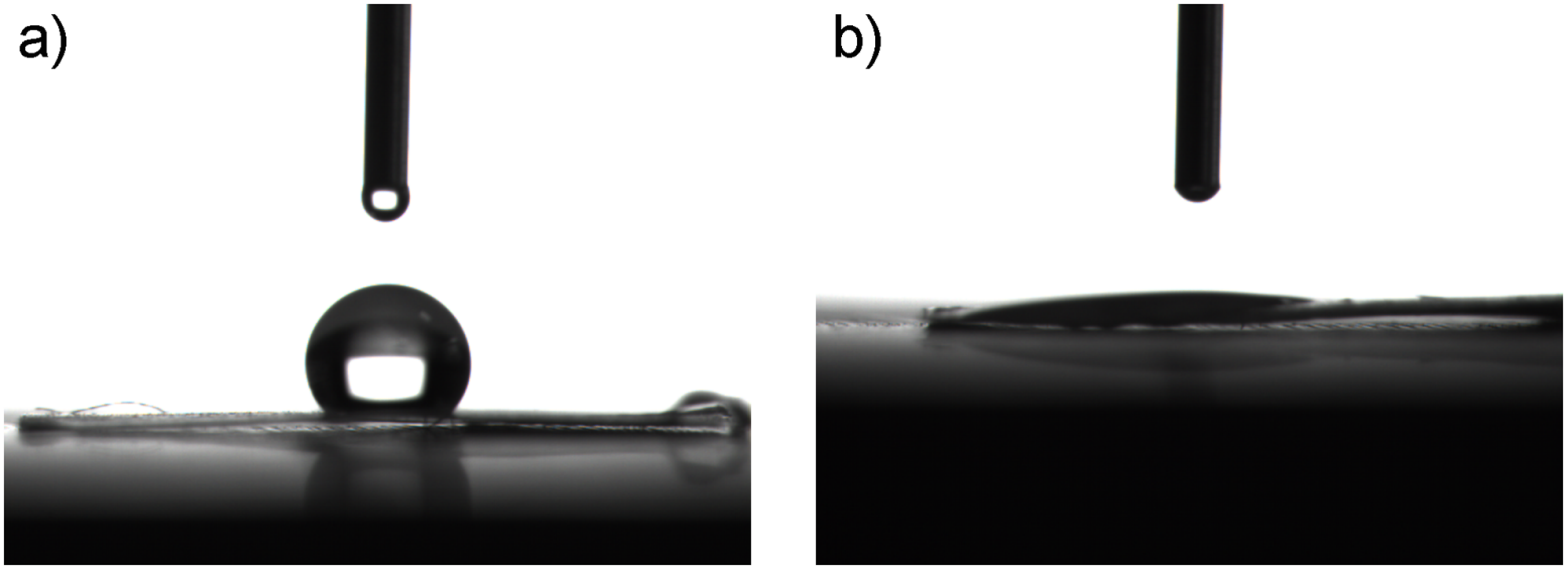
Contact angle test of nanofiber membranes. a) PCL membrane and b) NGFP membrane.

Fig 4 shows contact angles of NGFP nanofiber and PCL membranes. When one drop of water was added onto the surface of PCL membrane, a contact angle of 130° was detected, at which the drop could not spread out, indicating the surface of PCL membrane was hydrophobic (Fig 4a). While, water drop could easily spread out on NGFP membrane at a contact angle of 10° (Fig 4b), showing NGFP membrane had a hydrophilic surface. The results ratify that LBL assembling method is effective for modification of nanofiber membrane surface.

### 3.2 Preparation and Characterization of TMZ-doped PCL nanofiber membrane

**Fig 5:**
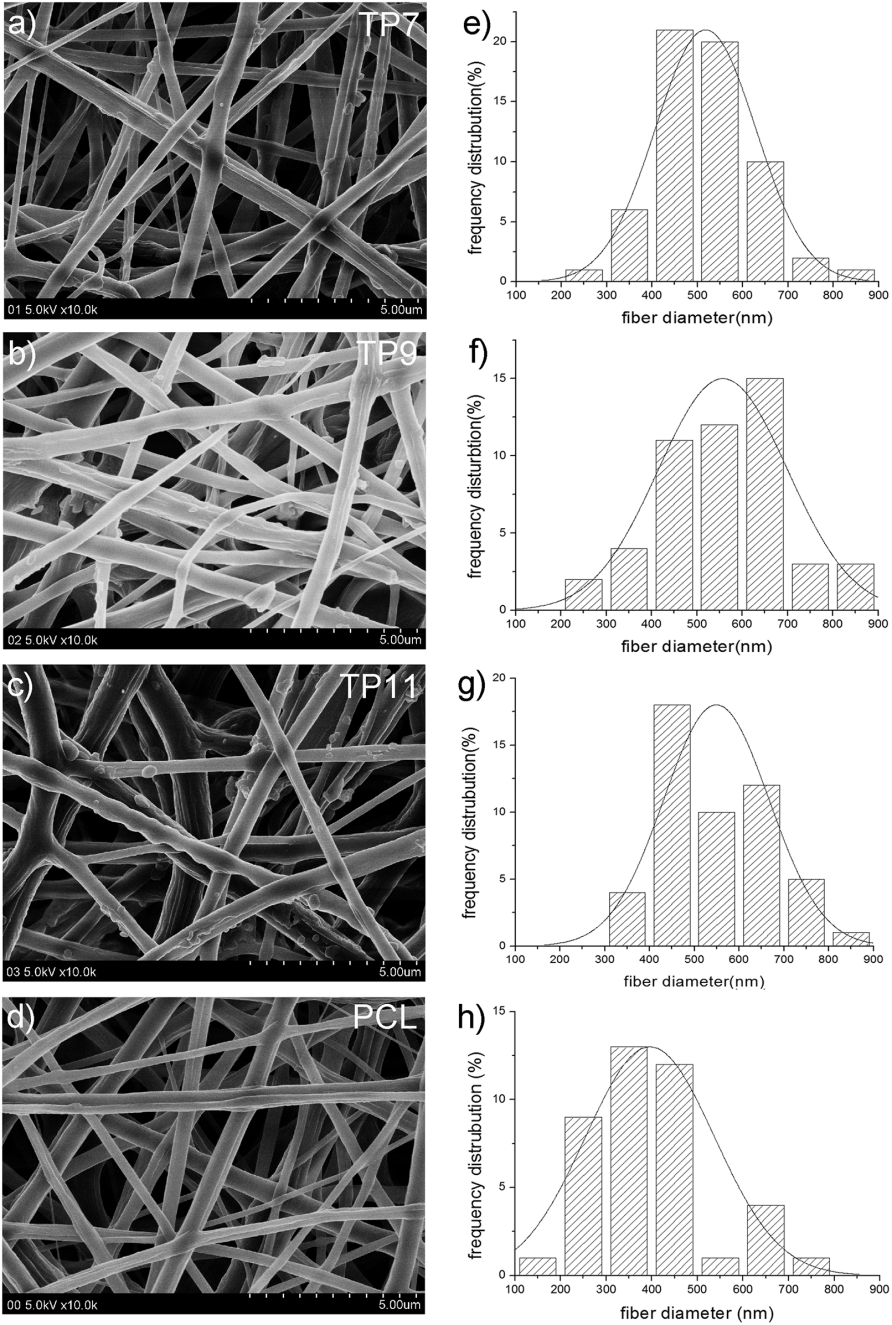
SEM images of TP membranes and their diameter distribution analysis. **PCL membrane is used as a control**. a) TP7, b)TP9; c) TP11; d) PCL; e-h) size distribution of TP7, TP9 TP11 and PCL, respectively.

TMZ is soluble in water and DMSO but not in non-polar solvent. As such, a mixed solution of PCL in DCM and TMZ in DMSO at different feed volume ratio was prepared to yield a homogenous medium for electrospinning. As a result, three TP membranes (*i*.*e*. TP7, TP9 and TP14) with different TMZ contents from 2.8 wt% to 7.2 wt% were prepared. TMZ contents of these membranes were relatively lower than theoretical values (Table 1). Fig 5a–5c exhibit typical images of these TP nanofibers observed under SEM. In general, these TP nanofibers displayed coarser surface compared to PCL nanofiber without TMZ (Fig 5d). Besides, they had similar average diameter (about 518–557 nm), which were slightly larger compared to that of parent PCL nanofibers (396 nm). The size distribution of TP7 nanofiber was more symmetry compared to that of other nanofibers, suggesting that TP7 nanofiber is relatively uniform in size. TP11 nanofiber displayed relatively coarse surface, likely due to its high TMZ content.

**Table 1:** Characteristics of TP membranes.

Code	Feed solvent ratio (DCM/DMSO)	Theoretical TMZ Content (wt%)	Tested TMZ content (wt%)	Mean diameter of nanofiber (nm)
TP7	7:3	7	2.8	518.1±111.1
TP9	6:4	9	3.9	557.3±142.7
TP11	5:5	11	7.2	549.0±117.8
PCL	-	-	-	396.0±141.3

### 3.3 TP-NGFP-TP membrane device for TMZ/NGF delivery

**Fig 6:**
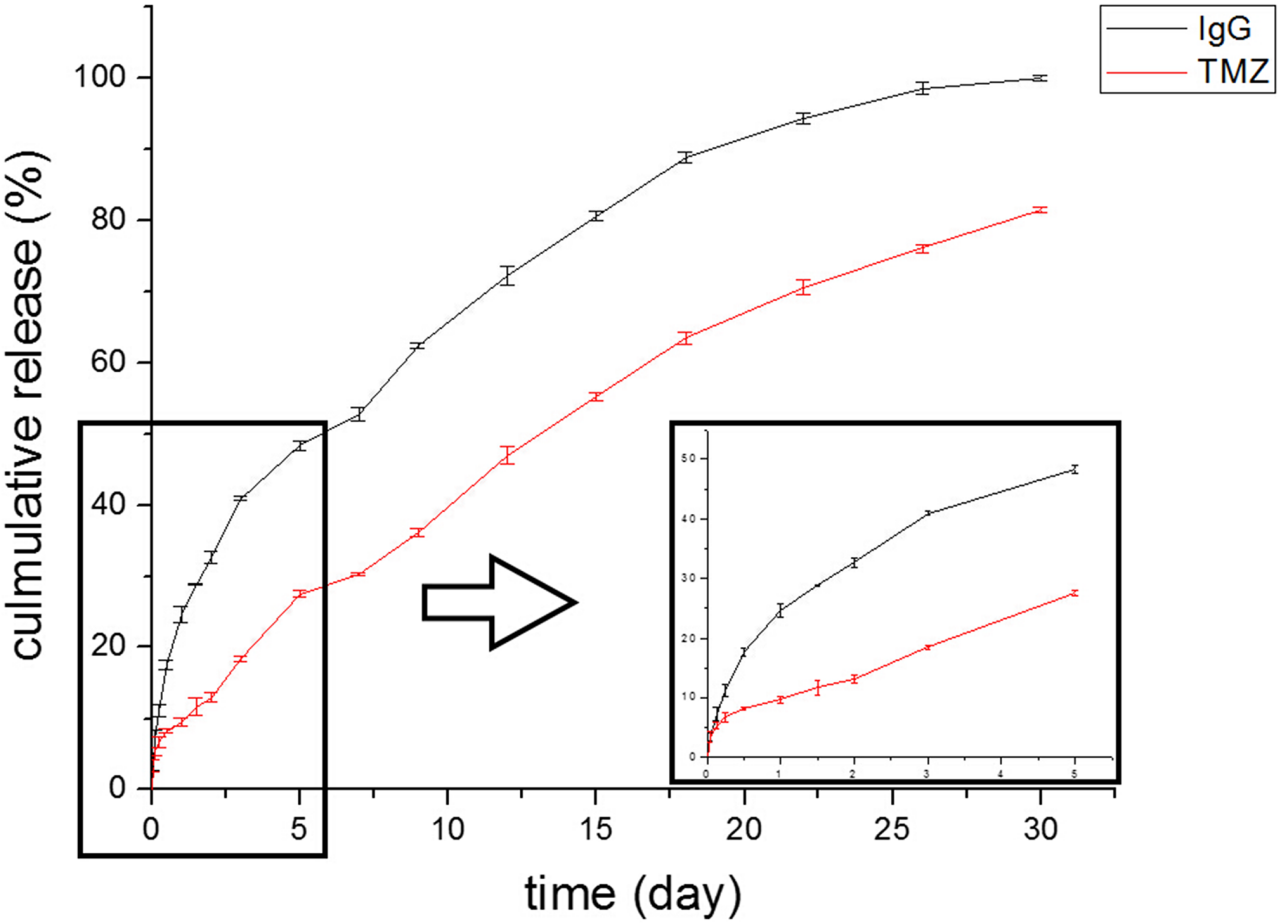
*In vitro* TMZ and protein release profile of TP7-IgGP-TP7 device constructed with TP7.

A membrane device releasing TMZ and NGF was constructed by coupling overlaid TP-NGFP-TP membrane sandwich with sodium alginate hydrogel. The hydrogel was used as a sticker to integrate TP membrane and NGFP membrane. The membrane TP7 with a high TMZ content was used to construct such sandwich device. TMZ release behavior of the device was investigated in PBS. As shown in Fig 6, the device could sustainably deliver TMZ during 30 days and ~78% of released TMZ was detected at day 30.

Fig 6 also shows the release feature of FITC-labeled IgG from TP7-IgGP-TP7 device, as determined by fluorescence density analysis. These results showed that the device was able to efficiently liberate protein in the period of 30 days, implying that such protein-coated membrane device should be useful for long-term protein release.

### 3.4 Anti-tumor effect of TP-NGFP-TP device

**Fig 7:**
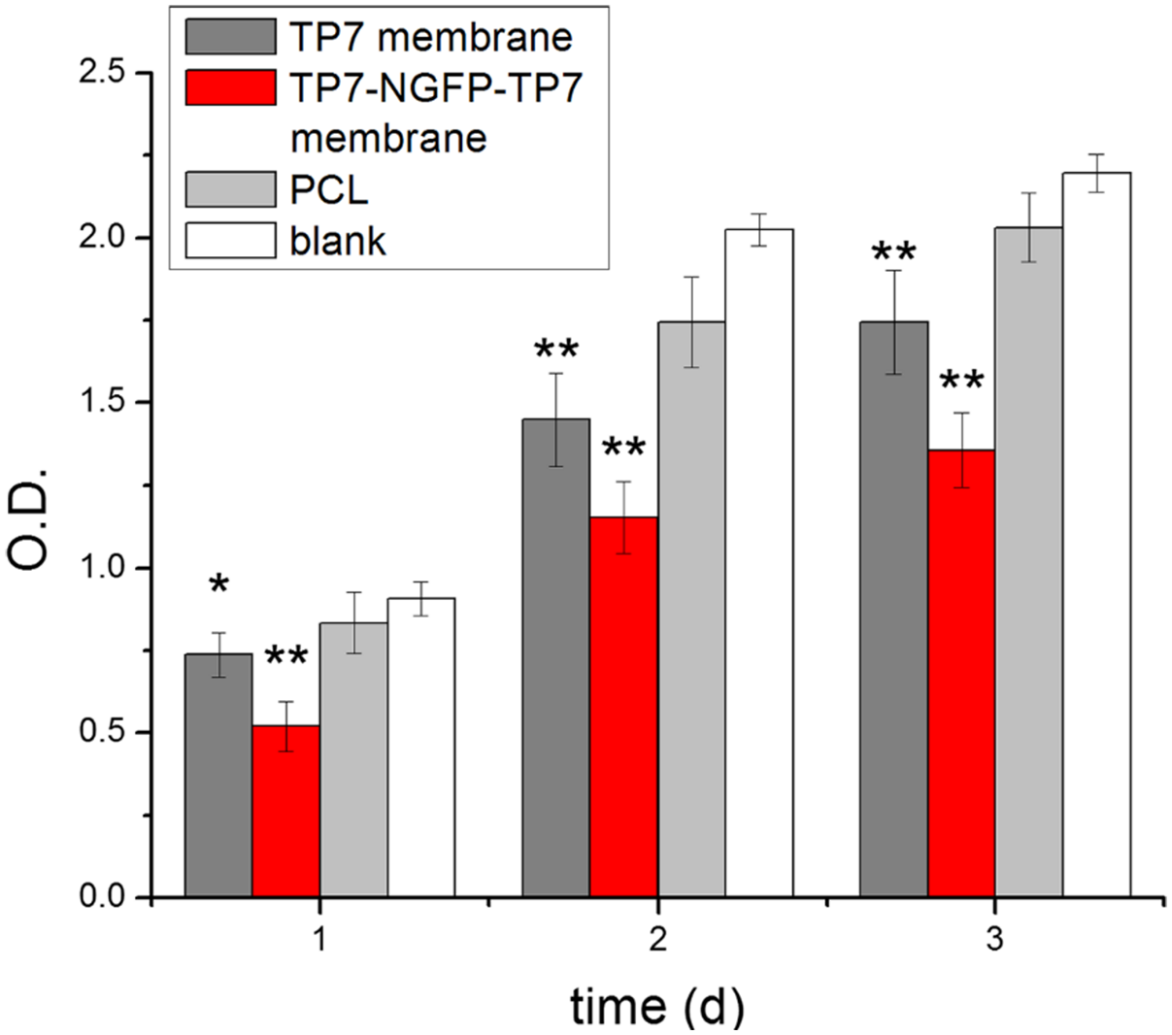
Anti-tumor effect of TP7-NGFP-TP7 device against C6 cells. **TP7 and PCL membrane were used as controls**. The blank indicates the cells without treatment. (*P<0.01; **P<0.001).

Anti-tumor activity of TP7-NGFP-TP7 device was performed by CCK-8 assay of C6 cell after co-incubating the cell with the device for 1–3 days. As shown in Fig 7, TP7-NGFP-TP7 device and TP7 membrane exerted significant growth inhibition of C6 cells to the blank control group after 3 days’ co-incubation. TP7-NGFP-TP7 device also afforded more pronounced cell growth inhibition as compared to TP7 membrane. A rational explanation is that TP7-NGFP-TP7 with two TP7 membranes has higher TMZ content than TP7 membrane.

**Fig 8:**
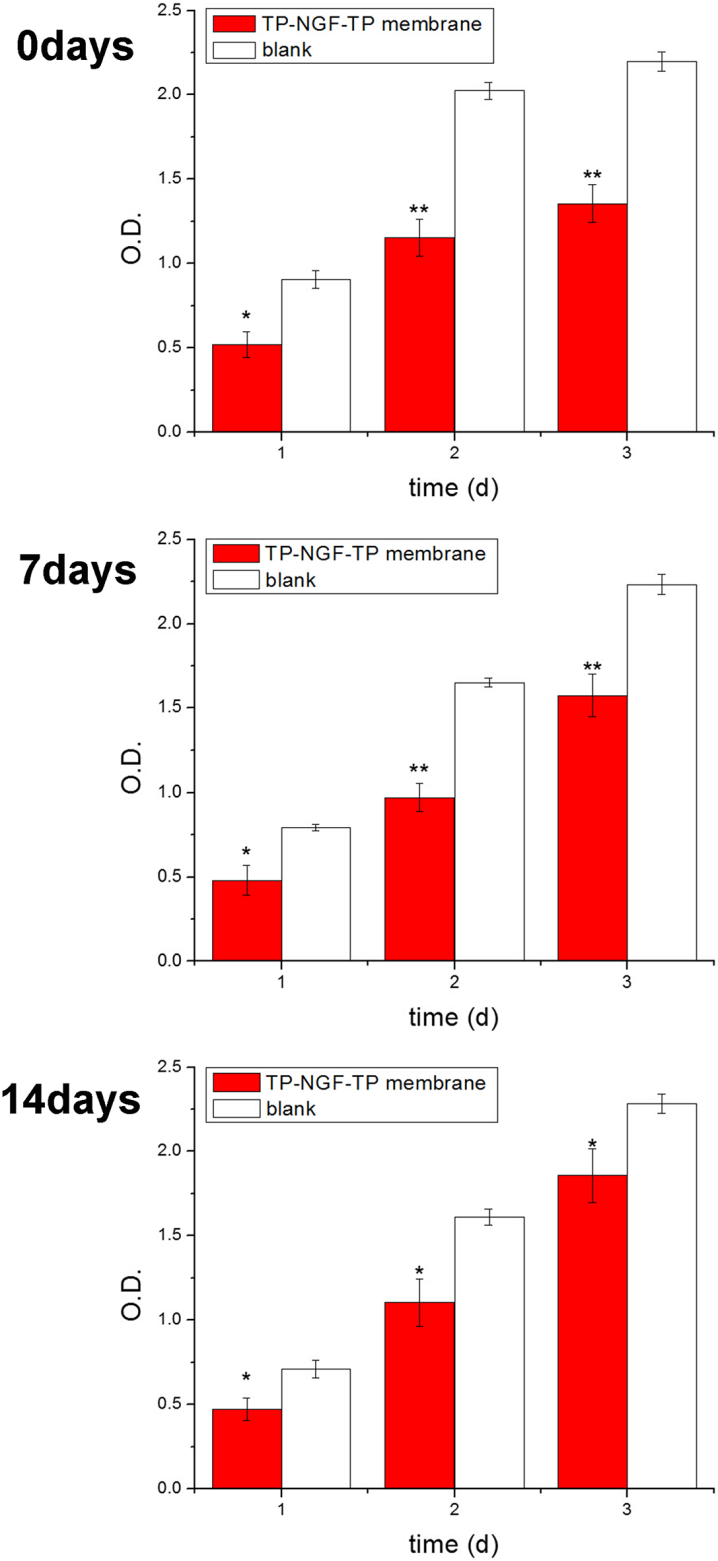
Anti-tumor effect of TP7-NGFP-TP7 device after pre-incubated for 0, 7 and 14 days in PBS. The blank indicates the cells without treatment. (*P<0.01; **P<0.001).

In order to assess anti-tumor ability of TP7-NGFP-TP7 device, it was pre-treated with PBS for different time (7 or 14 days) and its anti-tumor ability against C6 cells was detected. As shown in Fig 8, after pre-incubated in PBS (37°C, pH = 7.4) for 7 and 14 days, TP7-NGFP-TP7 device could inhibit the proliferation of C6 cells as compared to the blank group The *in vitro* tests indicated that the device may have the potential to suppress glioma growth.

### 3.5 The effect of NGFP membrane on neuron differentiation

**Fig 9:**
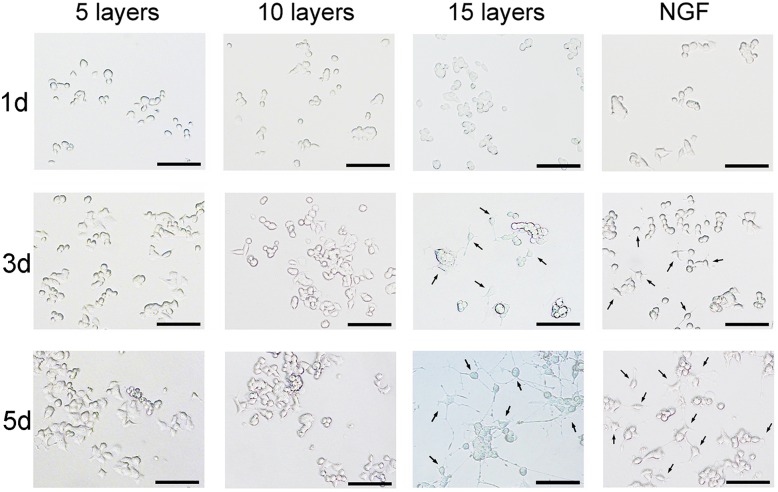
Microscope observation of PC12 cells after treated with NGFP membranes containing different NGF layers for 1, 3, 5 days. NGF at a concentration of 25 ng/mL was used as the positive control. The arrows indicate neuritis associating with differentiation of PC12 cells. The scale bar is 50 μm.

NGF is usually used to improve the prognosis of glioma in clinical trials [17–19], and dose not have the ability to promote the proliferation of C6 glioma cells (Fig B in S1 File). Also, NGF may induce the differentiation of PC12 cells into neuron-like cells (Fig C in S1 File) and thus used as the substitute for neuron cells in this work [20–22]. Thus, in order to evaluate the effect of TP-NGFP-TP device on the growth of neural tissue, PC12 cells were used in cell differentiation study as the differentiated PC12 cells are distinguishable under light microscope. NGFP membrane, rather than TP-NGFP-TP device, was directly applied in the preliminary *in vitro* test because TMZ released from TP-based device could kill PC12 cells. As shown in Fig 9, at the concentration of 25 ng/mL, the NGF induced pronounced differentiation of PC12 cells at day 5. A similar phenomenon was also observed for the 15-layer NGFP membrane, which induced sprout axon-like differentiated neuron cells. However, the NGFP membrane with 5- or 10-layers failed to induce the cell differentiation. Moreover, these membranes with less NGF layers appeared to afford shorter neurites compared to that with 15-layers. This result could be due to lower content of NGF in the culture for the membrane with 5- or 10-layers. In further test, to confirm the ability of TP7-NGFP-TP7 device to induce the differentiation of PC12 cells, TP7-NGFP-TP7 device with 15-layers was pre-incubated in PBS (pH = 7.4) at 37°C for 2 weeks, and the resultant device was then co-cultured with PC-12 cells. It was found that this device still retained the ability to induce the differentiation of PC12 cells (Fig D in S1 File). These results suggested that sufficient NGF layer, i.e. adequate NGF content, in NGFP membrane is critical to induce neural cell differentiation and in turn potential neural tissue reconstruction.

## Conclusions

In summary, we have developed a biodegradable multi-layer PCL membrane device loaded with TMZ and NGF. Such device plays a dual role in inhibiting *in vitro* growth of glioma cells and improving the differentiation of neuron cells. Such a membrane device could have high potential to be used as surgical residual cavity tampon to improve the local bioavailability of TMZ and NGF, exerting strengthened on site effects on prevention of glioma recurrence and metastasis, reduction of chemo side effect and acceleration of the local neural tissue reconstruction in the treatment of glioma. Subsequent *in vivo* studies are necessary to ascertain its practicability as a multi-function tampon for glioma surgery.

## Supporting Information

S1 File (A) EDS Data of NGFP membrane with different numbers of layers; (B) O.D. value of C6 cells cultured with NGF (50 ng/ml). Cells cultured with ordinary culture medium were set as the control; (C) Cellular morphologies of PC12 in cell cultures incubated with TP7-NGFP-TP7 device containing different NGF layers for 1, 3, 5 days. Devices were incubated in PBS (pH = 7.4, 37°C) for 14 days in advance; (D) Immunofluorescence images of PC12 cells cultured with TP-NGFP-TP and NGF (25 ng/ml) for 3 and 5 days. Cell nuclei stained blue (with DAPI) and β-tubulin III stained green (with AlexaFluor^®^488). Cells cultured with ordinary culture medium were set as the control.
(ZIP)
