# Supplementary figures and images for: A Potential Nanofiber Membrane Device for Filling Surgical Residual Cavity to Prevent Glioma Recurrence and Improve Local Neural Tissue Reconstruction
Source: PLoS One. 2016 Aug 22;11(8):e0161435. doi: 10.1371/journal.pone.0161435 (PMC4993477; doi:10.1371/journal.pone.0161435)

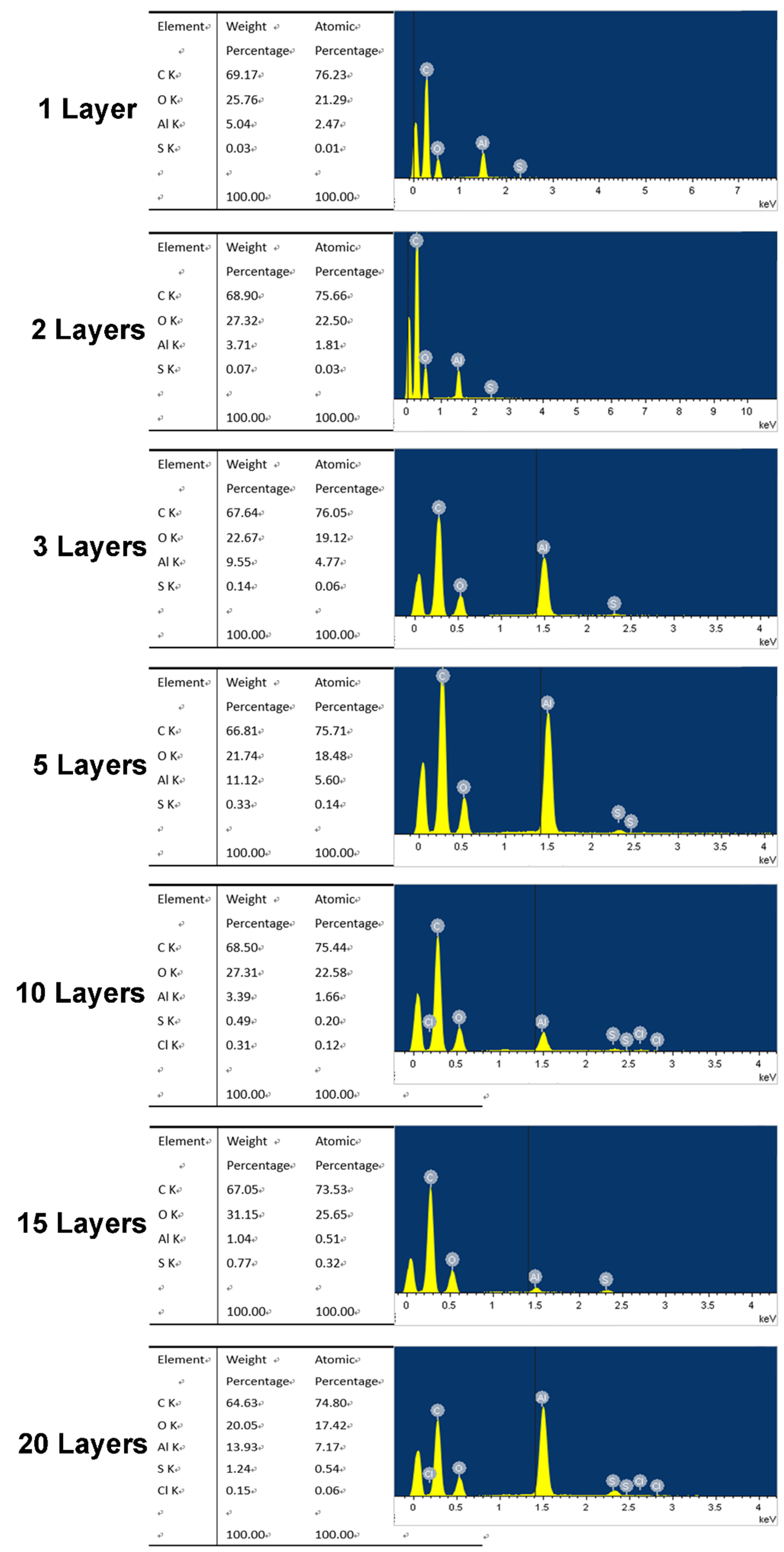

Supplement: S1 File — (A) EDS Data of NGFP membrane with different numbers of layers; (B) O.D. value of C6 cells cultured with NGF (50 ng/ml). Cells cultured with ordinary culture medium were set as the control; (C) Cellular morphologies of PC12 in cell cultures incubated with TP7-NGFP-TP7 device containing different NGF layers for 1, 3, 5 days. Devices were incubated in PBS (pH = 7.4, 37°C) for 14 days in advance; (D) Immunofluorescence images of PC12 cells cultured with TP-NGFP-TP and NGF (25 ng/ml) for 3 and 5 days. Cell nuclei stained blue (with DAPI) and β-tubulin III stained green (with AlexaFluor®488). Cells cultured with ordinary culture medium were set as the control. (ZIP) [file pone.0161435.s001.zip › Fig in Supporting information/Fig A in S1.tif]

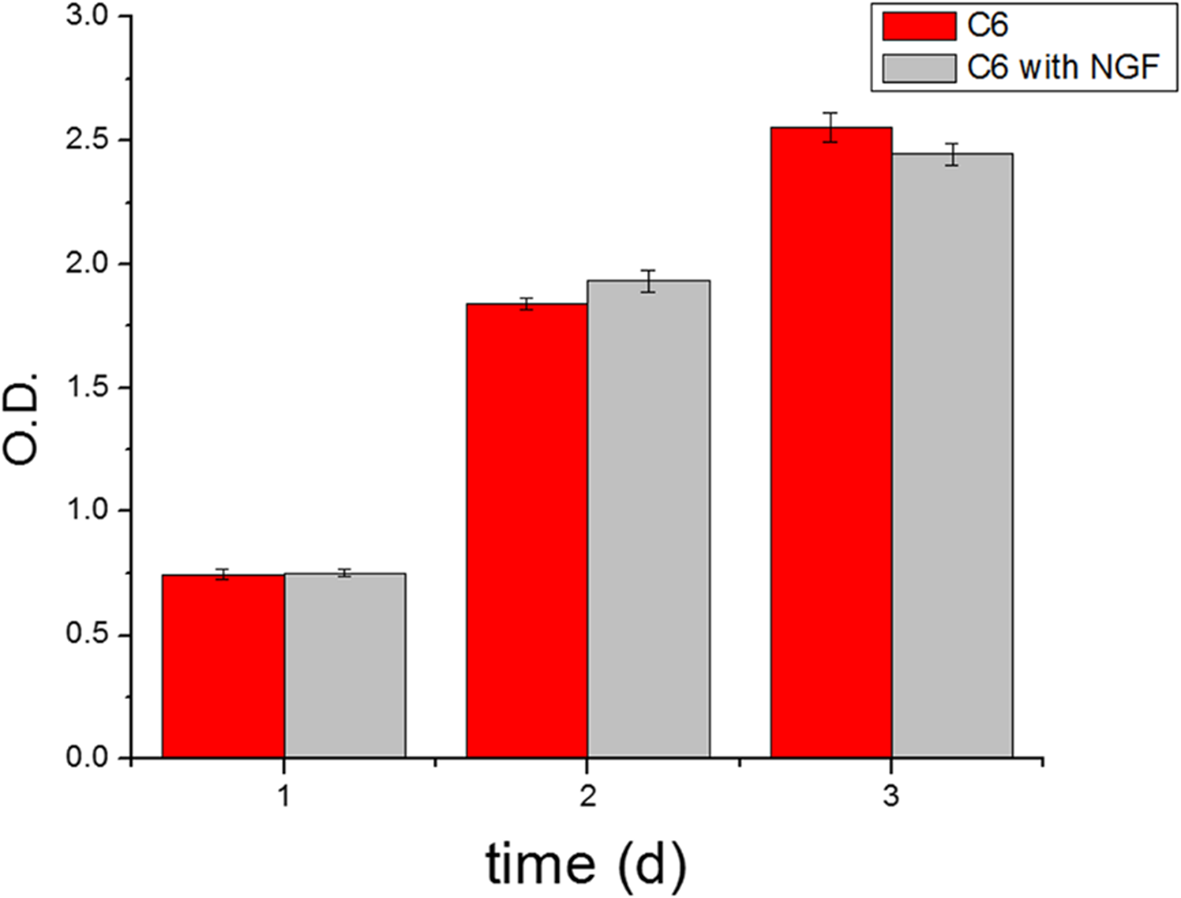

Supplement: S1 File — (A) EDS Data of NGFP membrane with different numbers of layers; (B) O.D. value of C6 cells cultured with NGF (50 ng/ml). Cells cultured with ordinary culture medium were set as the control; (C) Cellular morphologies of PC12 in cell cultures incubated with TP7-NGFP-TP7 device containing different NGF layers for 1, 3, 5 days. Devices were incubated in PBS (pH = 7.4, 37°C) for 14 days in advance; (D) Immunofluorescence images of PC12 cells cultured with TP-NGFP-TP and NGF (25 ng/ml) for 3 and 5 days. Cell nuclei stained blue (with DAPI) and β-tubulin III stained green (with AlexaFluor®488). Cells cultured with ordinary culture medium were set as the control. (ZIP) [file pone.0161435.s001.zip › Fig in Supporting information/Fig B in S1.tif]

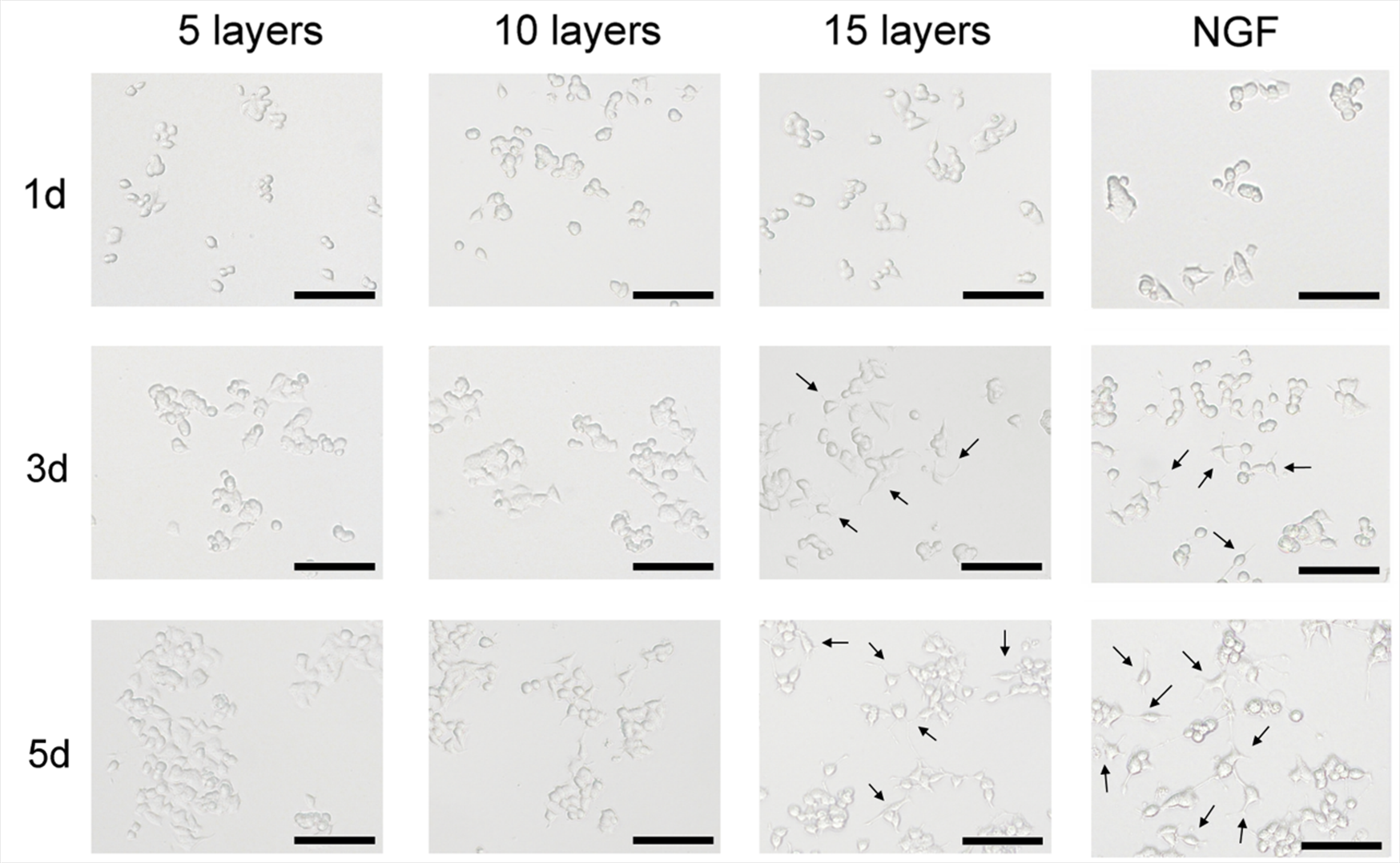

Supplement: S1 File — (A) EDS Data of NGFP membrane with different numbers of layers; (B) O.D. value of C6 cells cultured with NGF (50 ng/ml). Cells cultured with ordinary culture medium were set as the control; (C) Cellular morphologies of PC12 in cell cultures incubated with TP7-NGFP-TP7 device containing different NGF layers for 1, 3, 5 days. Devices were incubated in PBS (pH = 7.4, 37°C) for 14 days in advance; (D) Immunofluorescence images of PC12 cells cultured with TP-NGFP-TP and NGF (25 ng/ml) for 3 and 5 days. Cell nuclei stained blue (with DAPI) and β-tubulin III stained green (with AlexaFluor®488). Cells cultured with ordinary culture medium were set as the control. (ZIP) [file pone.0161435.s001.zip › Fig in Supporting information/Fig C in S1.tif]

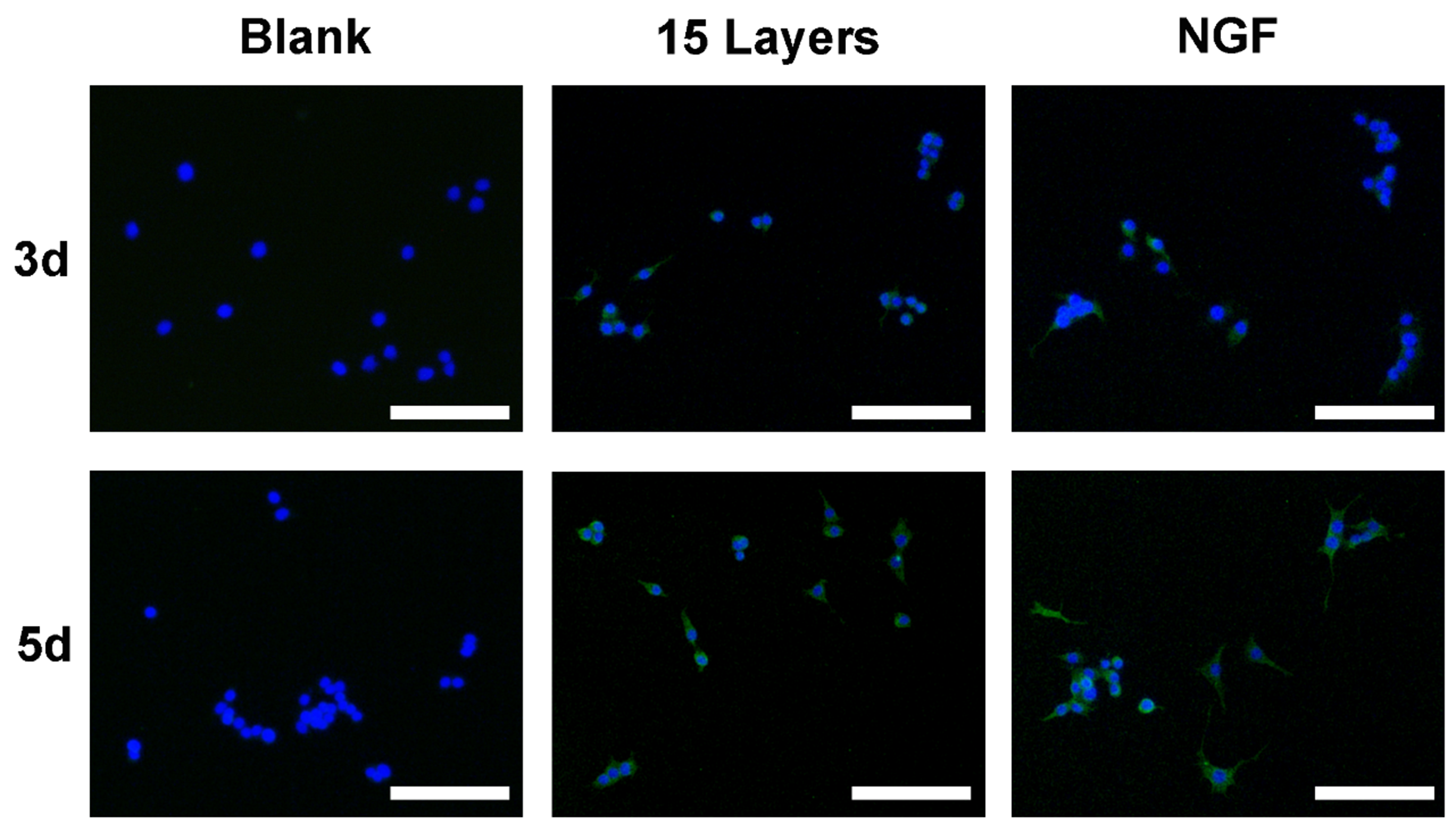

Supplement: S1 File — (A) EDS Data of NGFP membrane with different numbers of layers; (B) O.D. value of C6 cells cultured with NGF (50 ng/ml). Cells cultured with ordinary culture medium were set as the control; (C) Cellular morphologies of PC12 in cell cultures incubated with TP7-NGFP-TP7 device containing different NGF layers for 1, 3, 5 days. Devices were incubated in PBS (pH = 7.4, 37°C) for 14 days in advance; (D) Immunofluorescence images of PC12 cells cultured with TP-NGFP-TP and NGF (25 ng/ml) for 3 and 5 days. Cell nuclei stained blue (with DAPI) and β-tubulin III stained green (with AlexaFluor®488). Cells cultured with ordinary culture medium were set as the control. (ZIP) [file pone.0161435.s001.zip › Fig in Supporting information/Fig D in S1.tif]
